# Incidence and risk factors for pneumonitis due to trastuzumab Deruxtecan in metastatic breast cancer: a retrospective cohort study

**DOI:** 10.1186/s13058-025-02151-1

**Published:** 2025-11-05

**Authors:** Maria Azhar, Felipe Soto, Amber Su, Norma Alicia Vazquez Gonzalez, Kevin Bernal Medina, Alejandro Lizarraga Madrigal, Cesar Chavez Duran, Carlos Ignacio Rodriguez Reyna, Colin Chan, Girish S. Shroff, Roland L. Bassett, Sarah Pasyar, David Zhang, Vickie R. Shannon, Mehmet Altan, Melissa P. Mitchell, Funda Meric-Bernstam, Jason Mouabbi, Rashmi K. Murthy, Saadia A. Faiz, Lara Bashoura, Bora Lim, Ajay Sheshadri

**Affiliations:** 1 Divisions of Pulmonary, Critical Care and Sleep Medicine, McGovern Medical School, The University of Texas Health Science Center, Houston, USA; 2 Department of Pulmonary Medicine, Division of Internal Medicine, The University of Texas MD Anderson Cancer Center, 1515, Holcombe Boulevard, 77030 Houston, TX USA; 3 School of Medicine, Tecnológico de Monterrey, Monterrey, Mexico; 4 Texas A&M University School of Medicine, Houston, USA; 5 Department of Thoracic Imaging, Division of Diagnostic Imaging, The University of Texas MD Anderson Cancer Center, Houston, USA; 6 Department of Biostatistics, The University of Texas MD Anderson Cancer Center, Houston, USA; 7 Division of Pulmonary and Critical Care Medicine, Columbia University, New York, USA; 8 Department of Thoracic-Head & Neck Medical Oncology, Division of Cancer Medicine, The University of Texas MD Anderson Cancer Center, Houston, USA; 9 Department of Breast Radiation Oncology, Division of Radiation Oncology, The University of Texas MD Anderson Cancer Center, Houston, USA; 10 Department of Investigational Cancer Therapeutics, Division of Cancer Medicine, The University of Texas MD Anderson Cancer Center, Houston, USA; 11 Department of Breast Medical Oncology, Division of Cancer Medicine, The University of Texas MD Anderson Cancer Center, Houston, USA

## Abstract

**Background:**

Fam-trastuzumab deruxtecan (T-DXd) is an antibody-drug conjugate (ADC) that targets human epidermal growth factor receptor 2 (HER2) and delivers a topoisomerase inhibitor payload. T-DXd has been effectively used to treat metastatic breast cancer but causes pneumonitis in 10–15% of cases. Risk factors associated with T-DXd pneumonitis are not well described.

**Research question:**

What are the major clinical risk factors for T-DXd pneumonitis?

**Study design and methods:**

We conducted a retrospective study of women with metastatic breast cancer at our institution treated with T-DXd as standard of care between 2020 and 2024. We collected clinical data, including demographics, relevant review of symptoms, molecular subtypes, prior treatments, and pulmonary comorbidities, from the electronic health record. A thoracic radiologist reviewed pre-treatment scans for the presence of interstitial lung abnormalities (ILAs). Radiation exposure variables were obtained from an institutional database. Pulmonologists reviewed all cases for the development of pneumonitis. We used univariable and multivariable Cox proportional hazard models to measure the association of clinical variables with the development of pneumonitis.

**Results:**

Pneumonitis was observed in 19/203 patients (9.4%). In univariable analyses, pre-treatment shortness of breath (hazard ratio [HR] 3.9, 95% confidence interval [CI] 1.3–11.7), greater number of prior treatments (HR 1.2 per line, 95% CI 1.0-1.4), triple-negative breast cancer (HR 4.9, 95% CI 1.8–13.0), low HER-2 status (4.00 (95% CI: 1.43–11.11) and pre-treatment ILAs (HR 17, 95% CI 6.5–46) were associated with pneumonitis. In multivariable models, pre-treatment ILAs (HR 10.56, 95% CI 3.8-29.31, *p* < 0.0001) and HER2 (HR 0.30, 95% CI 0.09–0.96, *p* = 0.042) were the strongest predictors of pneumonitis, with a marginal association observed for V20 (HR 1.04, 95% CI 0.99–1.09, *p* = 0.081).

**Interpretation:**

Pre-treatment ILAs and HER2-low breast cancer are major risk factors for T-DXd pneumonitis.

**Supplementary Information:**

The online version contains supplementary material available at 10.1186/s13058-025-02151-1.

## Introduction

Trastuzumab-deruxtecan (T-DXd) has shown promising results in metastatic breast cancer, but initial studies have warned about potentially fatal cases of pneumonitis [1]. About 15–20% of breast cancers overexpress human epidermal growth factor receptor 2 (HER2) [2], resulting in dysregulated cell proliferation [3]. The development of the anti-HER2 monoclonal antibody trastuzumab marked a major breakthrough in the treatment of HER2 + breast cancers [4], but progression-free survival (PFS) was often measured in months [5]. The advent of antibody-drug conjugates (ADC) allowed for drugs to target HER2 overexpression in tumors as well as deliver a chemotherapeutic payload. The approval of the ADC fam-trastuzumab deruxtecan (T-DXd) marked a breakthrough in the care of HER2 + metastatic breast cancer, increasing PFS beyond one year [6]. Furthermore, T-Dxd has shown efficacy in HER2-low and -ultralow breast cancers, as well as solid tumors, thus expanding the indications for this targeted therapy [7, 8].

T-DX-d pairs trastuzumab with the topoisomerase I inhibitor deruxtecan. Prior studies of the topoisomerase I inhibitor irinotecan suggested that the combination of topoisomerase I inhibitors with other oncologic agents can increase the risk of pneumonitis, a potentially fatal pulmonary complication [9]. Pneumonitis has been observed in 10–15% of patients receiving T-DXd for advanced breast cancer in clinical trials [6, 8, 10, 11]. In pooled data, risk factors for pneumonitis included age less than 65 years, enrollment in Japan, lung disorders, moderate to severe renal impairment, more than 4 years since cancer diagnosis, dosage greater than 6.4 mg/kg every three weeks, and baseline oxygen saturation less than 95%^12^. Since study populations often differ from real world cohorts, it is not clear whether these risk factors are applicable to routine clinical practice [13].

We conducted a retrospective study of metastatic breast cancer receiving T-DXd to understand risk factors in real world patients. We focused on the possibility of subclinical interstitial lung abnormalities (ILAs) [14] that could progress to interstitial lung disease (ILD). Since ILAs may be unrecognized, we hypothesized that the detection of ILAs might be a significant predictor of T-DXd pneumonitis, especially given the increased risk for pneumonitis with lung comorbidities. In addition, we also wanted to assess the association of exposure to radiation involving the chest [15]. Through these analyses, we aimed to detect early risk factors to identify those at risk for T-DXd pneumonitis.

## Methods

### Study participants and clinical data collection

We identified women with metastatic breast cancer who were treated with T-DXd therapy between 2020 and 2024 and had at least 3 months of follow-up from the time of treatment initiation. We collected clinical, imaging, and laboratory data from the electronic health record, including demographics, tumor expression of HER2, estrogen receptor (ER) and progesterone receptor (PR), pre-treatment pulmonary symptoms, lung disorders, prior lines of therapy, and other relevant data. Thoracic radiation dosimetry was collected from an institutional database, specifically mean lung dose (the average dose in proportion to the total lung volume) and V20 (the percentage of healthy lung volume receiving at least 20 Gy). This study was approved by the MD Anderson Institutional Review Board (2023 − 0447). A dedicated thoracic radiologist reviewed pre-treatment computed tomography (CT) of the chest or positron emission tomography (PET)-CT images for pre-treatment ILAs as per Fleischner guidelines [16]. Abnormalities were only considered to be ILAs if they involved at least 5% of non-dependent lung regions.

## Study definitions

We reviewed imaging from all participants who developed new respiratory symptoms after T-DXd initiation. Pneumonitis was diagnosed by expert pulmonologists and a thoracic radiologist using clinical symptoms, chest imaging, relevant laboratory results (including microbiologic data) and response to treatment (steroids and/or antibiotics). Alternative diagnostic considerations included infectious pneumonia, pulmonary edema, or malignant disease progression. Pneumonitis was diagnosed if participants developed new respiratory symptoms and new opacities on thoracic imaging in a pattern consistent with pneumonitis, in the absence of clinical and/or microbiological evidence for respiratory infection, pulmonary edema or progressive metastatic disease. The radiological patterns for pneumonitis were classified as interstitial pneumonitis, organizing pneumonia, possible diffuse alveolar damage, or non-specific by a dedicated thoracic radiologist. Pneumonitis was graded according to Common Terminology Criteria for Adverse Events (CTCAE) version 5.0 [17]. In accordance with the ASCO CAP 2018 HER2 Testing for Breast Cancer Guidelines, cases with immunohistochemistry (IHC) scores of 0 or 1 + were not classified as HER2-positive, and fluorescence in situ hybridization (FISH) testing for these scores was not considered. For cases with 2 + or 3 + IHC, HER2 status was determined through concurrent evaluation of gene amplification and protein overexpression [18].

### Statistical analysis

We constructed univariable and multivariable Cox proportional hazard models to measure the association of variables of interest with the outcome of pneumonitis. All variables with *p* < 0.1 were entered into the multivariable model, which was then refined to maximize Harrel’s concordance. We constructed extended Cox proportional hazard models with pneumonitis as a time-dependent variable to measure the association of pneumonitis with all-cause mortality. To illustrate the survival of patients with and without pneumonitis, we estimated mortality using the Kaplan-Meier method from the time of treatment initiation to the time of death or last follow-up. All statistical analyses were performed using R version 4.3.2.

## Results

### Characteristics of the study cohort

We identified 203 women with metastatic breast cancer who were treated with T-DXd. Table 1 summarizes the characteristics of the study cohort. The median age was 55 years. Because enrollment in Japan was associated with a higher incidence of T-DXd pneumonitis [12], we characterized ethnicity as Asian and non-Asian, and 17 patients were of Asian origin (8.4%). History of tobacco use was reported in 45 (22.1%) patients. Immune checkpoint inhibitors (ICIs) had been used prior to T-DXd treatment in 15 patients (7.4%). Only 8 patients had evidence of pre-treatment ILAs (3.9%). Supplemental Table 1 details the ILA patterns. The median follow up time was 443 days [IQR 321–650]. The median number of cycles received was 9 [IQR 4–14]. There was no statistically significant difference between those who developed pneumonitis and those who did not (*p* = 0.65).

Patients were divided into HER2 positive (101 patients, 50%) and HER2 low (63 patients, 32%). Other receptor characteristics included ER positive in136 patients (67%) and PR positive in87 patients (43%). Lung metastasis prior to T-DXd was present in 88 patients (44%). No patients were rechallenged with T-DXd after pneumonitis.

**Table 1:** Clinical and demographic characteristics of cohort

Characteristic	*N* (%)
Patients	203
Age (median [IQR])	55.00 [47.00, 65.00]
Asian ethnicity	17 (8.4)
People who previously smoked	45 (22.2)
Cough*	15 (7.4)
Shortness of breath*	23 (11.3)
Respiratory infection*	7 (3.4)
History of autoimmune disease	33 (16)
History of lung disease	26 (12.8)
Interstitial lung abnormalities	8 (3.9)
ER positive	136 (67.0)
PR positive	87 (42.9)
HER2 positive	101 (49.7)
HER2, by immunohistochemistry	
1+	65 (32)
2+	64 (31)
3+	74 (37)
Triple negative breast cancer	22 (10.8)
Metastasis to lung	88 (43.6)
History of ICI*	15 (7.4)
Prior treatment lines (median [IQR])	4.00 [3.00, 6.00]
Radiation therapy*	131 (64.5)
V20 (median [IQR])	14.00 [10.00, 17.00]
Mean lung dose (MLD, Gy) (median [IQR])	7.00 [5.18, 10.07]

*Prior to Trastuzumab Deruxtecan, IQR, interquartile range; ER, estrogen receptor; PR, progesterone receptor; HER2, human epidermal growth factor receptor 2; ICI, immune checkpoint inhibitor; ; V20, lung volume receiving ≥ 20 Gy

### Pneumonitis

19 participants developed pneumonitis (9.4%), and grades were as follows: 6 with grade 1 (32%); 7 with grade 2 (37%); 5 with grade 3 pneumonitis (26%); 1 participant with grade 4 (5%). No fatal cases of pneumonitis were observed. The median time to onset of pneumonitis was 179 days [IQR: 84.5–234.5]. Radiological outcomes varied, with full resolution of pneumonitis observed in 4 cases (21%) and partial resolution in 6/19 cases (32%). In the remaining 9/19 cases (47%), there was no appreciable radiological improvement from pneumonitis. Among the 19 cases of pneumonitis, 18 were adjudicated from chest CT and 1 was adjudicated from PET-CT.

## Risk factors for pneumonitis

Table 2 shows univariable predictors for pneumonitis. History of autoimmune disease (Hazard ratio [HR] 3.4, 95% confidence interval [CI] 1.3–8.5), ER/PR negative with low HER2 (ER-/PR-, HR 4.9, 95% CI 1.8–13.0), baseline shortness of breath prior to T-DXd (HR 3.9, 95% CI 1.4–10.8), greater lines of therapy prior to T-DXd (HR 1.2 per line, 95% CI 1-1.4), prior ICI treatment (HR 3.9, 95% CI 1.3–11.7) and baseline ILAs (HR 17.3, 95% CI 6.5–45.7) were associated with an increased risk for pneumonitis. We found no association of CDK 4/6 inhibitor therapies with pneumonitis, but these were more commonly given for HER2-low cancers (*p* < 0.001). HER2 expression (HR 0.25, 95% CI 0.1–0.7) was associated with a decreased risk for pneumonitis; the highest risk of pneumonitis was seen in those who had 1 + expression by immunohistochemistry (IHC), when compared to 3 + expression (HR 6.0, 95% 1.6–22). There was no association of primary tumor volume with pneumonitis.

Table 3 shows multivariable risk factors for pneumonitis. In multivariable analysis, pre-treatment ILAs were strongly associated with an increased risk of pneumonitis (HR 10.56, 95% CI 3.80–29.31, *p* < 0.0001). Additionally, HER2 positivity was linked to a reduced risk of pneumonitis (HR 0.30, 95% CI 0.09–0.96, *p* = 0.042). A marginal association was observed for V20, though it was not statistically significant (HR 1.04, 95% CI 0.99–1.09, *p* = 0.081). Harrel’s concordance for this model was 0.82.

**Table 2:** Univariable predictors for pneumonitis

Variable (*N* = 203)	Univariable HR (95% CI)	*p*-value
Age	1.02 (0.99–1.06)	0.216
Asian ethnicity	0.67 (0.09–4.99)	0.692
People who formerly smoked	2.08 (0.82–5.29)	0.123
Cough*	1.79 (0.41–7.74)	0.438
Shortness of Breath*	3.89 (1.40–10.83)	0.009
Respiratory Infection*	1.51 (0.20–11.34)	0.686
History of Lung Disease	0.72 (0.17–3.12)	0.662
History of Autoimmune Disease	3.36 (1.32–8.54)	0.011
Baseline ILAs	17.28 (6.54–45.65)	< 0.0001
ER Positive	0.70 (0.28–1.73)	0.434
PR Positive	1.53 (0.62–3.78)	0.352
HER2/neu Receptor Positive	0.25 (0.09–0.70)	0.009
HER2/neu Receptor, by IHC		
3+	Ref	
2+	2.33 (0.56–9.78)	0.246
1+	5.95 (1.65–21.6)	0.006
Triple negative disease	4.89 (1.84–12.97)	0.001
Lung Metastasis at Baseline	1.45 (0.58–3.67)	0.428
Prior Lines of Treatment	1.20 (1.03–1.40)	0.023
Use of CDK 4/6 inhibitor therapy*	1.40 (0.57–3.45)	0.47
Use of ICI*	3.86 (1.28–11.65)	0.016
ICI within 6 months of T-DXd	3.01 (0.4–22)	0.28
ICI within 3 months of T-DXd	4.57 (0.6–34)	0.14
Chest Radiation Prior to T-DXd-All	1.71 (0.61–4.74)	0.306
Total Lung V20 (%)-All	1.05 (1.00–1.10)	0.073
Mean Lung Dose (Gy)-All	1.03 (0.98–1.07)	0.284

*prior to Trastuzumab deruxtecan

HR, hazard ratio; CI, confidence interval; IQR, interquartile range; ILA, interstitial lung abnormalities; ER, estrogen receptor; PR, progesterone receptor; HER2, human epidermal growth factor receptor 2; IHC, immunohistochemistry; TDx, Trastuzumab Deruxtecan; V20, lung volume receiving ≥ 20 Gy.

**Table 3:** Multivariable predictors for pneumonitis

Variable (*N* = 203)	Hazard Ratio (HR)	95% Confidence Interval	*p*-value
HER2 positive	0.30	0.09–0.96	0.042
ILA	10.56	3.80–29.31	< 0.0001
V20	1.04	0.99–1.09	0.081

HR, hazard ratio; CI, confidence interval; IQR, interquartile range; HER2, human epidermal growth factor receptor 2;ILA, interstitial lung abnormalities; V20, lung volume receiving ≥ 20 Gy

## Association of pneumonitis with mortality

**Fig. 1:**
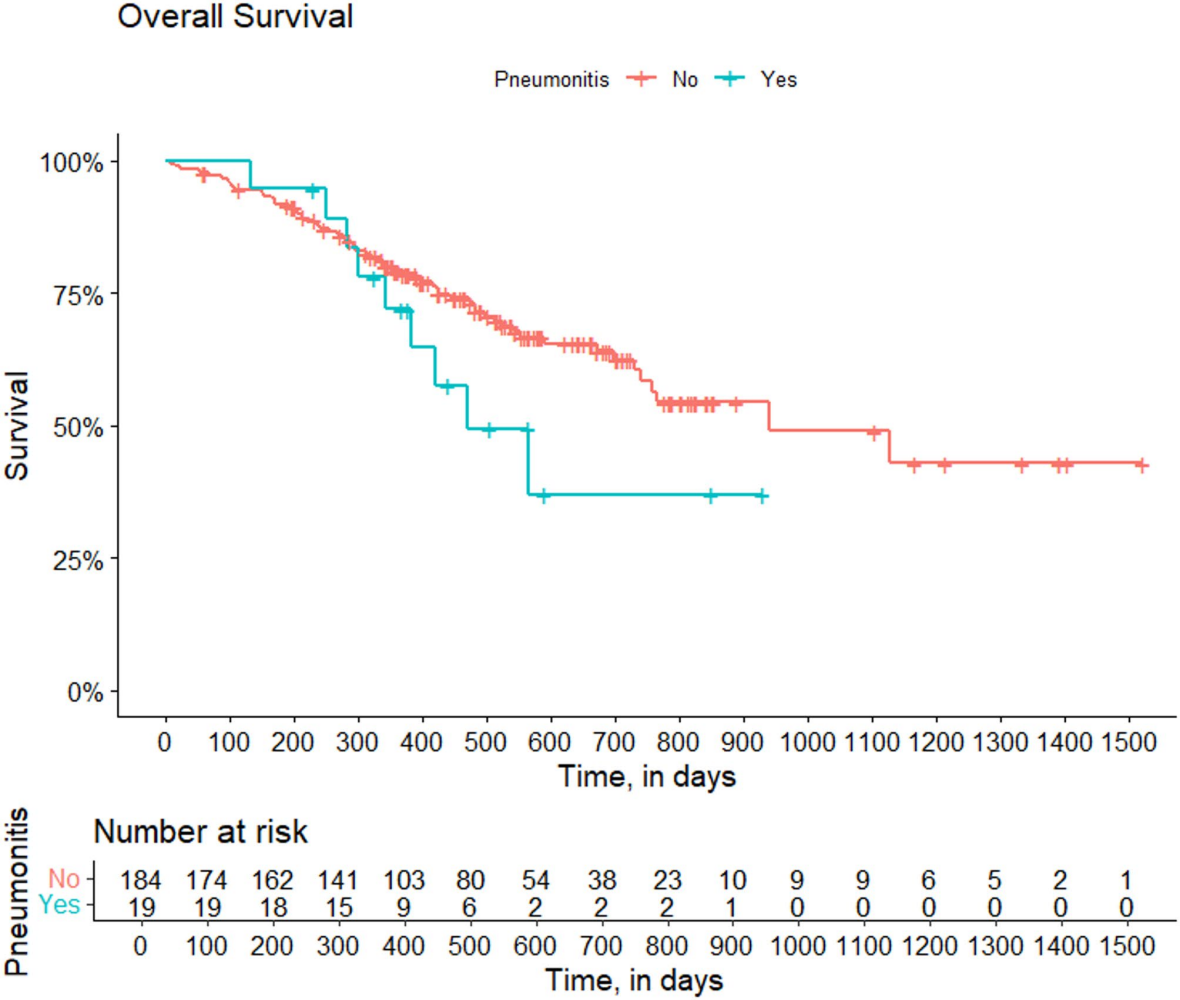
Overall survival stratified by pneumonitis

The median overall survival for patients who developed pneumonitis was 277 days, the median for those who did not was 940 days. We constructed extended Cox models to measure the association of pneumonitis with all-cause mortality. The risk associated with pneumonitis was only measured from the time of pneumonitis onset; in other words, in participants who developed pneumonitis, the period prior to pneumonitis diagnosis was not counted towards any association of pneumonitis with mortality. Though we saw no fatal cases of pneumonitis directly causing deaths, the development of pneumonitis was associated with increased mortality (HR 2.5, 95% CI 1.2-5.0). However, this association was attenuated after adjustment for ER/PR negative with low HER 2 status (HR 1.9, 95% CI 0.9-4.0). Figure 1 illustrates the increased mortality associated with pneumonitis, and the separation of the curves is more evident after 1 year following T-DXd therapy.

## Discussion

Our study illustrates that ILAs, while uncommon, are a major risk factor for the development of pneumonitis after T-DXd therapy in women with metastatic breast cancer. Surprisingly, pneumonitis was more common in those with ER/PR negative with low HER2 and low HER2 expression, suggesting off-target delivery of the deruxtecan payload may contribute to the risk for pneumonitis. After adjusting for other factors, we also observed an association of the mean lung radiation dose with pneumonitis. Our findings highlight risk factors for for T-DXd pneumonitis in a real-world cohort.

The main finding of our study is that prior ILAs were the strongest risk factor for T-DXd related pneumonitis. ILAs can represent an early form of idiopathic ILD, but even in the case of fibrotic ILAs, 40% may never progress [19]. The most common idiopathic ILD, idiopathic pulmonary fibrosis (IPF), is not characterized by excessive alveolar inflammation, as was once thought, but instead by accelerated aging of the alveolar epithelium [20]. IPF has been linked to polymorphisms in the gene MUC5B, leading to overexpression of that mucin and an increased innate immune response [21], as well as shortened telomeres, which are linked to cellular senescence [22]. ILAs and ILD are more common in people who smoke [23], and we have previously shown that they also increase the risk for ICI-associated pneumonitis in patients with lung cancer [24]. Since breast cancer is not as tightly linked to tobacco smoke as lung cancer, our lower incidence of ILAs in this cohort compared to lung cancer cohorts was expected. Despite this low prevalence, the presence of pre-treatment ILAs was the strongest predictor of pneumonitis. The Fleischner Society guidelines focus exclusively on thin-section and high-resolution CT for the identification and adjudication of ILAs and do not address PET-CT as a modality. Given that PET-CTs are often the only chest imaging obtained in some breast cancer patients, the Society should weigh in on how incidental ILAs seen on PET-CT ought to be interpreted. Other lung comorbidities were not associated with pneumonitis, suggesting that fibrotic lung diseases likely predispose to T-DXd pneumonitis [12]. The mechanisms whereby ILAs predispose to pneumonitis and other lung toxicities after cancer therapies remains unclear, but one possibility is that the alveolar epithelium in individuals with ILAs may be more vulnerable to damage [25].

It is plausible that HER2 expression by the primary tumor and potentially in the lung tissue may be intrinsically and mechanistically linked to the development of pneumonitis. While HER2 expression and its therapeutic implication have been thoroughly studied in the context of breast cancer, HER2 is also ubiquitously expressed in many other epithelial tissues, including the lung airways and alveoli [26]. Cigarette smoking increases HER2 expression in the lung while increasing epithelial permeability in preclinical models [27]. Dysregulation of the ERBB-YAP axis in the lung results in abnormal airway remodeling and promotes fibroblast activity to increase fibrosis [28]. HER2 activation in invasive fibroblasts is also associated with pulmonary fibrosis [29]. Thus, the risk of pneumonitis could also be potentiated by the presence of subclinical fibrosis in the form of ILAs. Our data demonstrates that lower HER2 expression by the primary tumor increased the risk for T-DXd pneumonitis suggesting the possibility that the risk for pneumonitis might be higher in HER2-low breast cancer due to more off-target delivery of the deruxtecan payload [30]. In fact, patients with ER/PR negative with low HER 2 had more than a 6-fold increase for pneumonitis. One may hypothesize that the same amount of drug concentrated in the lung environment with fewer HER2-expressing cancer cells, may instead target normal epithelial cells in the tumor microenvironment (TME) that also express HER2, thereby causing pneumonitis. Alternatively, the tumor microenvironment of triple negative breast cancer (which is similar to our ER/PR negative with low HER2 patients) is also characterized by heightened inflammation, potentially increasing the risk for toxicities, particularly with metastatic disease in the lung, but we did not find an association between lung metastases and pneumonitis [31–33].

Since HER2 inhibition alone is not associated with a significant risk for pneumonitis [4, 34], and the risk for pneumonitis is much lower with other ADC payloads [35, 36], it is likely that the deruxtecan portion of T-DXd primarily contributes to the risk for pneumonitis. Further studies are needed to validate this proposed mechanism. Our work also shows that pneumonitis is an independent predictor of late mortality, even though no patients developed fatal pneumonitis. Early pivotal trials of T-DXd suggested that about 1 in 6 cases of pneumonitis had fatal outcomes [6]. It is not clear why our incidence of fatal pneumonitis was lower, but earlier studies likely heightened the vigilance for toxicity among treating clinicians [37]. Despite this, among patients who developed pneumonitis, we found that only 21% had full resolution of radiological opacities, and in nearly half of cases, there was no appreciable improvement despite therapies. Moreover, late mortality may also be attributed to ineligibility for further therapies, though an important possible confounder is the association of HER2-low disease. Though our analyses adjusted for HER2 expression, it is possible that residual confounding exists, and the late mortality is more reflective of underlying risk due to HER2-low disease.

Two studies other than ours have examined risk factors for pneumonitis due to T-DXd. In a study of 1150 participants enrolled in 9 clinical trials, of whom 44% had breast cancer, age less than 65 years, enrollment in Japan, dose greater than 6.4 mg/kg, baseline oxygen saturation (SpO_2_) less than 95%, moderate/severe renal impairment, lung comorbidities, and more than 4 years since cancer diagnosis were associated with a higher risk for pneumonitis [12]. We found no association between most of these factors and the risk for pneumonitis, though we defined hypoxemia as SpO_2_ less than 92%, which has the strongest association with mortality [38]. If an SpO_2_ is in the normal range, it is not clear whether it is appropriate to use to determine if a patient is at high risk for T-DXd pneumonitis. Additionally, it is not clear whether the pulmonary disorders in the pooled analyses reflected ILD or other diseases, though our work suggests that it is primarily related to ILAs. Some of these differences may be because our findings are limited to individuals with advanced breast cancer receiving a dose of 5.4 mg/kg. In a study focusing on 179 individuals with breast cancer receiving T-DXd, the primary predictor for pneumonitis was abemaciclib therapy (38). We found no association with CDK 4/6 inhibitors and pneumonitis, though most CDK 4/6 inhibitors were given for ER/PR negative with low HER2 cases. Interestingly, the study by Henricks and associates found no association with prior ILAs and pneumonitis, but nearly 50% of individuals had evidence of prior chest radiological abnormalities, far higher than reported in healthy cohorts [23]. As with all retrospective studies, these findings require prospective validation.

Our study has several strengths. First, we consecutively enrolled all eligible patients at our institution. Second, pneumonitis was diagnosed by experienced pulmonologists after careful consideration of other causes for pulmonary impairment. Third, all pre-treatment scans were reviewed by a dedicated thoracic radiologist with significant expertise in ILAs and ILD. Fourth, because our study focused on readily available clinical data as well as use of electronic medical record, there was minimal missing data. Several limitations also exist. First, this is a retrospective study, and therefore we cannot rule out the possibility of unmeasured confounding factors, even in adjusted analyses. Second, though ILAs were the primary risk factor for T-DXd pneumonitis, only 5% of individuals had ILAs, and therefore these findings need to be replicated in a larger cohort. Third, the diagnosis of ILAs is optimal on dedicated chest computed tomography, but in many cases, we only had CT images from PET-CT available to us. Fourth, we did not have genomic data to investigate the mechanisms whereby ILAs increased the risk for pneumonitis. Fifth, we had a relatively low incidence of pneumonitis, which limits out ability to fit multivariable models. However our findings represent the largest series to date, and it suggests that the risk for pneumonitis may be predictable in a real-world cohort but requires prospective validation.

## Conclusion

We found that the primary risk factors for T-DXd pneumonitis were prior ILAs, ER/PR negative, and HER2-low disease. Some of these findings may also be relevant to other cancers, particularly since T-DXd now has an indication for the treatment of all HER2-overexpressing solid tumors [39]. In particular, the risk for pneumonitis is highest in HER2-high non-small cell lung cancer [40], and people who smoke have a higher risk for ILAs and ILD [23]. Further mechanistic studies are needed to understand whether HER2 expression in the lung increases the risk for T-DXd pneumonitis, and whether other ILA or ILD risk markers may be relevant to predict individuals at high risk for T-DXd pneumonitis.

## Supplementary Information

Below is the link to the electronic supplementary material.

Supplementary Material 1

## Data Availability

Data is available upon request.
