# Supplementary material for: Incidence and risk factors for pneumonitis due to trastuzumab Deruxtecan in metastatic breast cancer: a retrospective cohort study
Source: Breast Cancer Res. 2025 Nov 5;27:197. doi: 10.1186/s13058-025-02151-1 (PMC12587553; doi:10.1186/s13058-025-02151-1)
Supplement: Supplementary file 1 — Supplementary Material 1 [file 13058_2025_2151_MOESM1_ESM.docx]

**Supplemental Table 1.**Pattern of ILAs

| **Pattern** | **Cases (n)** | **Proportion (out of 8)** |
| --- | --- | --- |
| Basilar reticulation without honeycombing | 3 | 3/8 |
| Subtle atelectasis or scarring | 1 | 1/8 |
| Isolated ground glass opacities | 1 | 1/8 |
| Ground glass opacities with reticulation | 1 | 1/8 |
| Subpleural ground glass opacities with reticular opacities | 1 | 1/8 |
| Subpleural ground glass opacities and reticulation in the lower lobes, with radiation therapy-related changes | 1 | 1/8 |
